# Resilient Multi-Sensor UAV Navigation with a Hybrid Federated Fusion Architecture

**DOI:** 10.3390/s24030981

**Published:** 2024-02-02

**Authors:** Sorin Andrei Negru, Patrick Geragersian, Ivan Petrunin, Weisi Guo

**Affiliations:** School of Aerospace, Transport, and Manufacturing, Cranfield University, Cranfield MK43 0AL, UK; patrick.geragersian@cranfield.ac.uk (P.G.); i.petrunin@cranfield.ac.uk (I.P.); weisi.guo@cranfield.ac.uk (W.G.)

**Keywords:** UAV, urban air mobility, computer vision, multipath, resilient navigation, hybrid fusion, GRU, EKF

## Abstract

Future UAV (unmanned aerial vehicle) operations in urban environments demand a PNT (position, navigation, and timing) solution that is both robust and resilient. While a GNSS (global navigation satellite system) can provide an accurate position under open-sky assumptions, the complexity of urban operations leads to NLOS (non-line-of-sight) and multipath effects, which in turn impact the accuracy of the PNT data. A key research question within the research community pertains to determining the appropriate hybrid fusion architecture that can ensure the resilience and continuity of UAV operations in urban environments, minimizing significant degradations of PNT data. In this context, we present a novel federated fusion architecture that integrates data from the GNSS, the IMU (inertial measurement unit), a monocular camera, and a barometer to cope with the GNSS multipath and positioning performance degradation. Within the federated fusion architecture, local filters are implemented using EKFs (extended Kalman filters), while a master filter is used in the form of a GRU (gated recurrent unit) block. Data collection is performed by setting up a virtual environment in AirSim for the visual odometry aid and barometer data, while Spirent GSS7000 hardware is used to collect the GNSS and IMU data. The hybrid fusion architecture is compared to a classic federated architecture (formed only by EKFs) and tested under different light and weather conditions to assess its resilience, including multipath and GNSS outages. The proposed solution demonstrates improved resilience and robustness in a range of degraded conditions while maintaining a good level of positioning performance with a 95th percentile error of 0.54 m for the square scenario and 1.72 m for the survey scenario.

## 1. Introduction

The emergence of the UAM (urban air mobility) concept necessitates more stringent requirements and regulations to ensure safe operations between manned and unmanned vehicles within the same airspace. Authorities such as EASA (European Union Aviation Safety Agency), CAA (Civil Aviation Authority), and FAA (Federal Aviation Administration) have already established specific requirements for regulating the air traffic in urban, semi-urban, and rural environments for UAVs, as specified in [1,2,3]. In this context, to ensure the safety of operations in urban environments, the PNT solutions provided by UAVs operating in the proximity of buildings and obstacles must be continuous, robust, and resilient. In addition, as it can be seen from [4], UAVs can play a key role in multi-spectral mapping applications and other civil applications [5], where the need for a stable PNT system is crucial to fulfilling all the mission requirements.

Given that the GNSS receivers serve as the primary source of PNT data for UAVs, which can provide good accuracy in open-sky conditions as presented in [6], where an RTK (real-time kinematics) system is implemented, external disturbances can quickly degrade their accuracy. Due to the nature of urban and semi-urban environments, NLOS (non-line-of-sight) and multipath signal propagation can decrease the quality of the GNSS signals, leading to erroneous localization. As the GNSS receivers are low-powered, low-cost jamming devices can easily emit electromagnetic interference over the same frequencies used by the GNSS receivers, resulting in an untrustworthy PNT solution. Spoofing is another threat affecting the PNT integrity, where false GNSS signals are broadcasted to deliberately degrade the PNT data. Thus, considering all the potential threats to the GNSS receivers, A-PNT (alternative position, navigation, and timing) sensors should be used to achieve better navigation performance, even when the GNSS is not able to provide a reliable PNT solution.

An IMU serves as an A-PNT sensor, typically formed by three accelerometers and three gyroscopes, providing data regarding the linear acceleration and angular velocity of the carrier in each direction of the body frame. Usually, UAVs are equipped with MEMS (microelectromechanical system) IMU sensors to derive the position and attitude using an INS (inertial navigation system) mechanization process. Unfortunately, the INS mechanization leads to positioning drift over time as specified in [7,8], making the IMU unreliable when used in a standalone mode for long flight operations.

A-PNT sources, including sensors such as stereo or monocular optical cameras, are alternative methods of improving positioning accuracy and precision in situations when the GNSS is unavailable. Motion estimation for optical A-PNT sources can be classified into two categories: RVL (relative visual localization) or AVL (absolute visual localization). RVL is computed through the application of VO (visual odometry) [9,10] and SLAM (simultaneous localization and mapping) algorithms, as presented in [11,12,13]. VO methods involve the analysis of the frames captured by an optical sensor to estimate its motion through the environment. Instead, the SLAM (simultaneous localization and mapping) approach represents a more intricate navigation algorithm capable of estimating the relative motion of the UAV while simultaneously building the surroundings on the map. Therefore, VO can be regarded as a subset of SLAM-based navigation. However, it is important to note that in challenging environments characterized by low light conditions or scarcity of distinctive features, both VO and SLAM algorithms can cause divergence in their motion estimation due to drift. Instead, for estimating the UAV’s absolute position, the VPS (visual positioning system) algorithm can be used, as presented in [14,15]. To successfully implement the VPS, it is essential to have a proper dataset with georeferenced aerial images that covers the AoI (area of interest). Additionally, the tilt angle of the camera during the flight should align with the used dataset in order to enhance its overall performance. Positioning accuracy can be affected by additional factors, such as seasonal changes and the ongoing construction of new buildings and roads, potentially causing mismatches with the dataset in use. Therefore, updated and recurrent datasets are required.

Although it is possible to extract PNT information from various sensors, a fusion approach is required in order to combine all the advantages offered by each A-PNT sensor. Multi-sensor fusion frameworks can be categorized into either CF (centralized fusion) or DF (decentralized fusion) frameworks. Even if a CF architecture can provide a reliable PNT solution, its high computational cost can lead to the so-called ‘computational disaster’ effect, as described in [16]. To mitigate computational costs and enhance the robustness of the fusion framework, a DF approach can be used. Instead of using only one filter, as in the CF architecture, the DF framework implements multiple local filters in parallel, fusing their output into a final master filter. Thus, the computational cost can be divided among all the local filters, and multiple A-PNT sensors can be added easily as subsystems. This method results in a federated DF framework suitable for real-time applications, as described in [17], where KFs (Kalman filters) were implemented. While the FF (federated fusion) architecture with KFs demonstrates good performances, it is important to note that in the real world, A-PNT sensors are susceptible to external noise, as discussed in [18], which can negatively impact the accuracy of KFs. In addressing non-linear systems, various solutions such as EKFs (extended Kalman filters), UKFs (unscented Kalman filters), and PFs (particle filters) have been widely used before, as specified in [19]. Their effectiveness is contingent upon prior knowledge of the measurement noise and processing noise.

Instead, fusion frameworks based on RNNs (recurrent neuronal networks) have demonstrated good performance in modeling and predicting the behavior of A-PNT sensors in real-world testing scenarios, as presented in [20], by fusing the INS and GNSS to cope during GNSS outages. This approach has demonstrated a notable 60% improvement against a traditional EKF (extended Kalman filter). However, there are certain drawbacks to using RNNs, including their high computational cost and the challenge of long-term data storage, which can introduce errors over extended periods of time. Moreover, if the weights are too small, the learning rate becomes slow, and managing data over time can decrease the performance of the RNN, leading to the so-called ‘vanishing gradient’ effect. Conversely, if the weight is too large, the output can diverge, leading to an ‘exploding gradient’ effect. Hence, to improve the performance of RNNs, LSTM (long short-term memory) and GRUs (gated recurrent units) introduce gates that aid in the longer-term memory capability of the RNN. As it can be seen in [21,22], LSTM models are used to enhance positioning accuracy in urban environments. Even better performances were obtained by implementing a GRU model to cope with GNSS outages, as presented in [23,24].

Thus, to assess the performance of combining GRUs with traditional fusion methods such as EKFs, this paper introduces a hybrid federated fusion architecture for 3D positioning. The federated architecture uses two EKFs as local filters and a GRU model as a master filter to predict the position of the UAV during a flight mission performed in an urban environment. The system gathers data from various A-PNT sensors, including a GNSS receiver, a MEMS IMU sensor, a monocular camera, and a MEMS barometer. To enhance the realism of the data collected, a HIL (hardware in the loop) set-up is used, which involves using Spirent’s GSS7000 simulator tools (SimGEN and SimSENSOR) along with OKTAL-SE (Sim3D) to gather GNSS data with multipath and MEMS IMU data. At the same time, a virtual environment in Unreal Engine is used to integrate a monocular camera to be used by a VO algorithm in order to estimate the UAV’s ego motion through the urban environment. In addition, the MEMS barometer readings from the virtual UAV in Unreal Engine are integrated into the federated fusion framework to cope with the instabilities introduced by the VO on the z axis during the flight mission. The paper’s key contributions can be summarized as follows:

1. The research introduces, in a three-dimensional scenario, a hybrid fusion architecture that integrates GRU (gated recurrent unit) and EKF (extended Kalman filter) systems. This study offers a detailed comparison of the new hybrid approach against the traditional FF (federated fusion) architecture.
2. To evaluate the performance of the proposed hybrid FF architecture using a range of realistic trajectories with the aim of mimicking real-world UAV operations, including multipath and GNSS outages.
3. To assess the influence of the optical part of the fusion algorithm by introducing various weather conditions, including dust and fog. In addition, the VO algorithm was tested during different light intensities, both in the afternoon and in the evening, using realistic photogrammetry data.

The reminder of the paper is structured as follows: In Section 3, the proposed hybrid federated fusion architecture is presented; in Section 4, the HIL configuration is detailed; in Section 5, the trajectories, the camera calibration steps, and the performances of the hybrid federated fusion architecture are discussed; and in Section 6, the conclusions and future work are given.

## 2. Related Works

Multi-sensor fusion frameworks have been widely used to assure a robust and resilient position and navigation for autonomous systems. With the advent of ML, hybrid fusion frameworks have been increasingly adopted in recent times by combining KFs and ML models. Standalone ML fusion models can be used without the integration of any KF, but their performance is limited when used with MEMS IMU sensors, as presented in [25]. The main disadvantage of implementing standalone ML models used to fuse GNSS and MEMS IMU sensors without KFs is represented by the absence of feedback to update the measurement model of the MEMS IMU inertial sensor, which is crucial due to its rapid change in dynamics over time. Thus, hybrid fusion methods can combine the advantages of KFs and ML models, which can be divided into three categories, as specified in [26].

In the first category, ML models are used as aids to tune KFs, as presented in [27], where a RBFN (radial basis function network) and a PSO (particle swarm optimization) are used as aids to cope with the non-linearities of the system. On the other hand, in [28], the authors developed a NN (neuronal network) as an aid to an AKF (adaptive Kalman filter) to adjust the system noise parameters.

Instead, in the second category, hybrid fusion methods are used in combination with ML models to predict INS errors, while GNSS signals are not available. As presented in [29], an UKF is used with a BP (back propagation) neuronal network to cope with GNSS outages. When the GNSS receiver is not affected by external disturbances, the BP model is trained using the position errors provided by the UKF as input, and when there are GNSS outages, the BP-trained model is used to enhance the positioning output by correcting the INS data. Although the solution proposed by the authors improves the position output during GNSS outages, the BP model has inferior performance compared to the UKF model during normal operations when the GNSS is available. Meanwhile, better results are presented in [30], where the authors implemented a GRU model along with an AKF to cope with GNSS outages. The GRU model is trained with GNSS data when available and used to predict GNSS position measurements during GNSS disturbances, measurements that are used as input for an AKF with INS data. Results showed a reduction in root mean square error of 83.03% and 75.39% during the 180 and 120 seconds of GNSS outages, respectively, proving the efficiency of the GRU-trained model in combination with an AKF. As a drawback, the solution presented by the authors considers only one scenario, and further data collection is required to better evaluate the presented hybrid fusion framework.

In the third category, ML models can be used to enhance fusion methods in combination with fault detection approaches for real-time applications and in complex environments. As presented in [31], a RBFNN (radial basis function neuronal network) is used to predict pseudo-GNSS measurements when faulty GNSS data is detected, aiming to improve fault isolation and system reconfiguration in a tightly coupled approach. The main challenge in the solution proposed by the authors is to optimally tune the POP (precision of positioning) and RDOP (relative differential precision of positioning) thresholds, which define the filter precision.

Hybrid fusion methods have been widely used to predict errors related to GNSS/IMU fusion configurations. Considering the complexity of urban environments, it is unlikely that UAVs will rely solely on GNSS and MEMS IMU sensors to cope with all the external disturbances. Hence, the proposed fusion framework is investigating the performance of a hybrid federated fusion framework in a complex urban environment, relying solely on a GNSS, a MEMS IMU, an optical camera, and a MEMS barometer.

## 3. Proposed Hybrid Federated Fusion Solution

**Figure 1:**
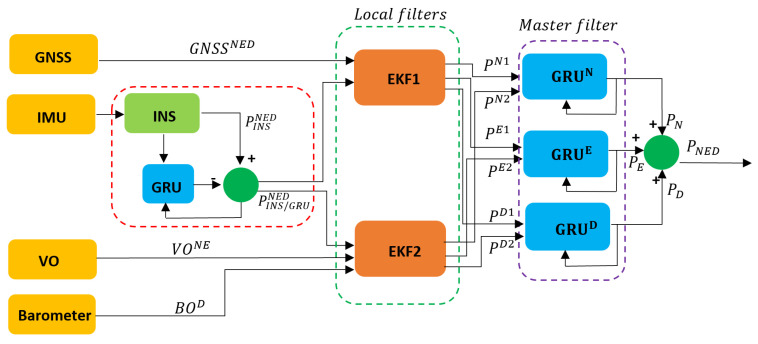
Proposed hybrid federated multi-sensor fusion architecture.

The proposed hybrid federated fusion architecture involves the fusion of four different sensors, formed by a GNSS receiver, a MEMS IMU sensor, a monocular camera, and a MEMS barometer sensor, to enhance the PNT solution, even in proximity to urban and sub-urban areas. The proposed fusion architecture adopts a hybrid approach, combining machine learning techniques using a GRU with traditional fusion architectures such as the EKF, as can be seen in Figure 1. The hybrid approach is used to improve the accuracy of the final positioning output, particularly when dealing with GNSS data affected by multipaths and outages.

### 3.1. Trained INS GRU

**Figure 2:**
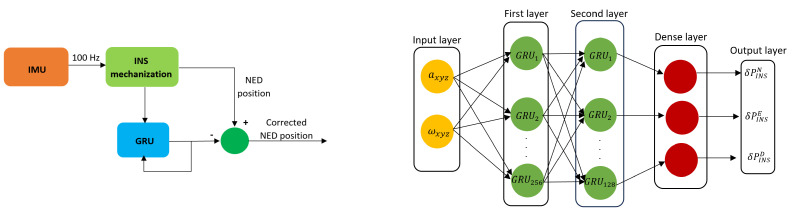
INS/GRU corrections—**left**; GRU diagram—**right**.

To cope with the INS drift over time, which leads to an erroneous position estimation, a GRU model is used to enhance the INS positioning output by predicting the INS behavior in time, as can be seen in the left part of Figure 2. During the training part, the GRU gathers raw MEMS IMU data as the input, formed by readings from the MEMS accelerometer and the MEMS gyroscope. As depicted in the right part of Figure 2, the input layer of the GRU block, which takes data from the provided dataset, is formed by the following components: *a* representing the linear acceleration and ω representing the angular velocity. For the training part, 80% of the dataset was used, while the other 20% was used for the testing part. As output, the GRU model provides INS corrections by comparing the estimated INS corrections against the ground truth, where ${\delta P}_{INS}^{N}$, ${\delta P}_{INS}^{E},\;and{\;\delta P}_{INS}^{D}$ represent the position error in the NED frame without using the input block called ‘time since last GNSS’, as presented in [24].

Once the GRU model has been trained, the estimated INS data are utilized to provide input to the federated fusion architecture, feeding the output into the two local filters, as can be seen in Equation (1). As a result, the impact of INS drift on the local filters diminishes over time.

(1) $$P_{INS/GRU}^{NED}=P_{INS}^{NED}-{\;\delta P}_{INS}^{NED}$$

### 3.2. GNSS/INS EKF

**Figure 3:**
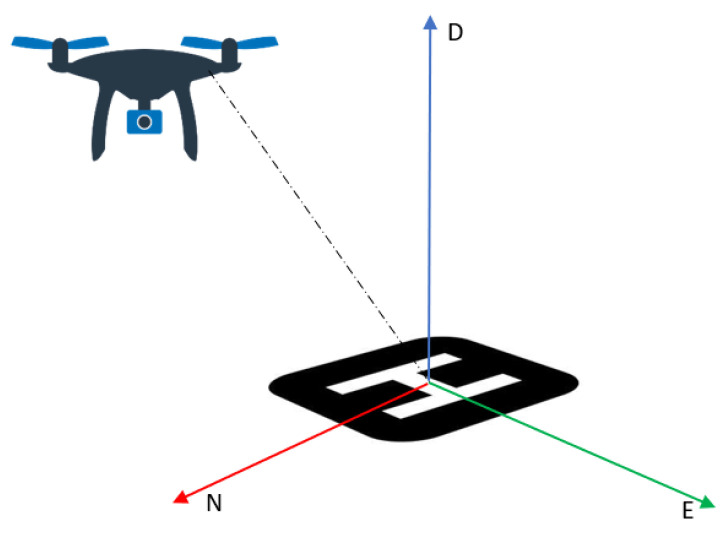
UAV in a NED frame.

The first local filter fuses data from the GRU block, presented in the previous section, and the GNSS in a loosely coupled approach. To fuse the output from the two sensors, both have to share the same navigation frame, as defined in Appendix A. Thus, GNSS data must be converted from the LLA (latitude, longitude, and altitude) frame to a NED coordinated frame as it can be seen in Figure 3. To set the conversion, an LLA reference base is defined as:

(2) $$\varphi_{REF}=43.604441{}^{\circ}\;\;\lambda_{REF}=1.4427133{}^{\circ}\;\;h_{REF}=0\;m$$

where $\varphi_{REF}$, $\lambda_{REF}$, and $h_{REF}$ are the initial altitude, longitude, and altitude, respectively. With the initial starting point coordinates, it is possible to realize the conversion from geodetic coordinates to geocentric coordinates. To begin, it is necessary to convert the data from the WGS84 (World Geodetic System 1984) to an ECEF (Earth-centered, Earth-fixed) coordinate system, as presented in [32]. After that, the ECEF position is converted to a NED coordinate frame, as specified in [33] and in Appendix A. Once the GNSS data is converted to NED coordinates, it can be fused with the GRU output. The state vector for the EKF is defined as follows:

(3) $$x_{k}=\left[\begin{array}{c} P_{xN}\\ P_{yE}\\ P_{zD} \end{array}\right]$$

The initialization step of the EKF is given by the UAV base position in the NED frame along the initial covariance matrix. Furthermore, the prediction step has the aim of estimating future states and is defined as follows:

(4) $${\hat{x}}_{k}^{-}=g\left({\hat{x}}_{k-1}^{-},u_{k-1}\;\right)\;{\hat{x}}_{k}^{-}={\hat{x}}_{k-1}^{-}F_{k-1}\;P_{k}^{-}=F_{k-1}P_{k-1}F_{k-1}^{T}+G_{k-1}Q_{k-1}G_{k-1}^{T}\;$$

where ${\hat{x}}_{k-1}^{-}$ is the initial UAV position, $u_{k-1}$ is the control input given by the INS GRU source, $F_{k-1}$ is the dynamic covariance matrix, $P_{k}^{-}$ is the priori covariance matrix, $G_{k-1}$ is the noise covariance matrix, and $Q_{k-1}$ is the process noise covariance matrix. The last step is represented by the update step (correction step). The number of states dictates the number of columns of the measurement matrix, and the number of measurements dictates the size of the rows. Once the measurement matrix is defined, the measurement residual, or innovation, can be defined as:

(5) $$y_{k}=z-h\left({\hat{x}}_{k}^{-}\right)$$

where $y_{k}$ is the measurement residual, z is the observation vector, and $h\left({\hat{x}}_{k}^{-}\right)$ is the measurement equation based on the predicted states. Once the measurement residual is obtained, the Kalman gain can be calculated, as defined in Equation (6). If its value is low (closer to 0), the predicted values are closer to the actual states; otherwise, the value will be closer to 1, meaning that the predicted values have more errors.

(6) $$K_{k}=P_{k}^{-}H_{k}^{T}{\left(H_{k}P_{k}^{-}H_{k}^{T}+R_{k-1}\right)}^{-1}$$

The measurement update from the GNSS is defined as:

(7) $$z_{GNSS_{k}}=\left[\begin{array}{ccc} 1 & 0 & 0\\ 0 & 1 & 0\\ 0 & 0 & 1 \end{array}\right]\left[\begin{array}{c} P_{xN}\\ P_{yE}\\ P_{zD} \end{array}\right]+\vartheta_{1,k}\;z_{GNSS_{k}}=\left[\begin{array}{c} P_{xN\_GNSS}\\ P_{yE\_GNSS}\\ P_{zD\_GNSS} \end{array}\right]$$

where $z_{GNSS\_k}$ is the measurement vector, $H_{GNSS}$ is the measurement matrix for the GNSS measurements, and $\vartheta_{1,k}$ is the Gaussian noise related to the measurements formed by a covariance matrix defined as $R_{k}^{GNSS}$. Finally, the predicted state vector and the predicted matrix are defined as:

(8) $$x_{k}={\hat{x}}_{k}^{-}+K_{k}\left(z-h\left({\hat{x}}_{k}^{-}\right)\right)\;P_{k}=\left(I-K_{k}H_{k}\right)P_{k}^{-}$$

### 3.3. INS/VO/Barometer EKF

The second local filter relies on positioning data from the trained INS GRU block and the positioning data generated by the VO algorithm along the MEMS barometer sensor. The VO algorithm is a visual-based technique widely implemented in robotics that can be used to estimate user motion from a sequence of images, especially when a GNSS solution cannot be provided. VO algorithms can be divided into two main categories: appearance-based and feature-based.

The appearance-based approach estimates the robot’s motion by analyzing pixel intensity information obtained from the output of an optical camera, as defined in [34]. Based on this approach, it is possible to derive two additional methods. The first method consists of using a template matching method, which can provide a motion solution by aligning two consecutive frames and measuring local unchanged similarities. The second method implements an optical flow algorithm that directly analyzes the changing intensity of pixels in two consecutive frames, computing a field of vectors from which motion can be estimated.

The second category is formed by feature-based methods, which do not track all the data from two consecutive frames but only key features such as lines or corners, which are effective in environments rich in details. From a computational point of view, the feature-based methods are more effective than the appearance-based methods.

Considering that urban and sub-urban environments are characterized mainly by a multitude of details, the authors implemented a feature-based approach.

Different feature-based algorithms can be implemented, each having different performances, such as the Harris-Corner detector [35], Shi-Tomasi corners [36], FAST (features from accelerated segment test) corners [37], SURF (speeded-up robust features) features [38], SIFT (scale invariant feature transform) features [39], and ORB (oriented FAST and rotated BRIEF) features [40]. In a more detailed analysis, as specified in [41], ORB shows the best performance in terms of computational load; thus, an ORB approach is chosen to deal with real-time missions. Furthermore, the ORB feature detector algorithm can be utilized without the requirement for a license. In contrast, other methods such as SIFT and SURF are subject to patents and, as a result, entail associated costs for usage.

The first step in using the ORB algorithm is to detect features using the FAST corners approach. The FAST algorithm begins by selecting a reference pixel to serve as the center and then considers all the pixels within a radius. After that, a threshold based on pixel intensity is calculated, and the position of features can be determined. Although the features can be detected, the FAST approach does not provide any direction information. Thus, as specified in [40], the intensity centroid (IC) approach is used to find and define the orientation vector, as specified in [42]. This solution increases the robustness of the detected features during rotatory movements, as specified in [40]. Furthermore, a steered BRIEF (binary robust independent elementary feature) descriptor is used, as defined in [40].

To match the descriptors between two frames, a matcher algorithm is needed. Thus, the FLANN (Fast Library for Approximate Nearest Neighbors) [43] is used for its real-time features and matching performance when many features occur against the BFmatcher algorithm.

After the feature matching step, a motion estimation method is needed to compute the ego motion of the camera, which is rigidly attached to the UAV. There are mainly three methods to estimate the ego motion of the camera, as follows [44]:

- 2D to 2D (both features are specified in 2D image coordinates between two frames)
- 3D to 3D (both features are specified in 3D image coordinates between two frames)
- 3D to 2D (previous features are specified in 3D coordinates and the current features in 2D image coordinates)

Considering that a single monocular camera is used, a 2D–2D method is adopted. Furthermore, the essential matrix is required in order to extract the ego motion of the camera, defined in [9] as follows:

(9) $$E={\left[t\right]}_{x}R$$

where R is the rotational matrix and t is the translational vector. From the estimated essential matrix, it is possible to extract the rotational matrix and the translation vector. Usually, four solutions are provided, but with triangulation, one single solution is extracting the ego motion of the monocular camera. In addition, to increase the accuracy of the estimated trajectory, the authors implemented the RANSAC (random sample consensus) algorithm, as specified in [45]. To utilize the VO data accurately, it is necessary to execute a conversion from the camera frame to the navigation frame, as specified in [46,47] and in Equation (A1).

(10) $$\left[\begin{array}{c} p_{t,N}^{n}\\ p_{t,E}^{n}\\ 1 \end{array}\right]={\lambda K}^{-1}{\left[\begin{array}{cc} T_{camera}^{NED}; & T_{camera}^{NED}r_{nc}^{n} \end{array}\right]}^{-1}\left[\begin{array}{c} u\\ v\\ 1 \end{array}\right]$$

where $T_{camera}^{NED}$ is the transformation from camera to the navigation frame, K is the intrinsic camera matrix, $r_{nc}^{n}$ is the position of the camera in the navigation frame, and λ is the scale factor. In comparison to the previous EKF presented in Section 3.2, a barometer is used to provide altitude information to cope with the instabilities provided by the monocular camera on the z axis. Thus, the N and E positioning coordinates are provided by the monocular camera using the VO algorithm, and the D positioning coordinate is provided by the barometer. Furthermore, the output from the VO and barometer is fused with the NED positioning output from the GRU model, which provides an enhancement of the INS output, as presented in Section 3.1.

### 3.4. Master Filter

**Figure 4:**
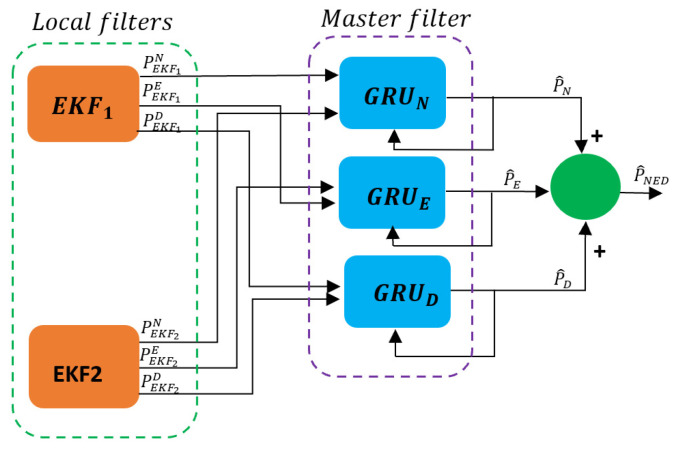
Master filter architecture.

The final section of the fusion framework is represented by three GRU models, used to enhance the output from the two EKFs as depicted in Figure 4. Since only the N, E, and D positions are considered, the first GRU model is used to process only the N position data, while the second and third GRU models are used to process only the E and D position data, respectively.

Each GRU model is formed by a layer consisting of 128 GRUs, a RELU activation layer, and a dense layer, followed by the final output layer. Each GRU model was trained using 80% of the dataset and tested using the remaining 20% of the dataset. After the training phase, the final output is defined as follows:

(11) $$P_{NED}={\hat{P}}_{N}+{\hat{P}}_{E}+{\hat{P}}_{D}$$

## 4. Hardware in the Loop Configuration

**Figure 5:**
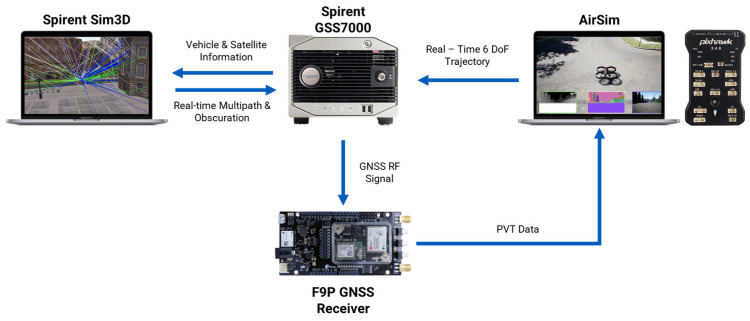
HIL set-up.

To enhance the realism of the dataset used by the hybrid sensor fusion architecture, a HIL configuration is established, as can be seen in Figure 5. For the HIL set-up, a Pixhawk 2.4.8 board is used and configured in the HIL mode by running the px4fmu-v2_default firmware, which represents the main FCU (flight control unit) of the UAV responsible for the navigation, control, and stability of the flying device.

Furthermore, once the connection is established with the hosting computer, the Pixhawk is calibrated properly by using the QGroundControl interface. After the calibration step, on the same hosting computer, Unreal Engine 4.27.2, Cesium v2.0.0, and AirSim 1.7.0 are launched with the HIL configuration file (required by AirSim), starting the HIL simulation. Unreal Engine is a 3D graphics interface that can be used to model specific simulation environments, such as urban, semi-urban, and rural environments. The hosting computer is equipped with an Intel Keon CPU E5-1650 v4, 32 GB of RAM, and an NVIDIA GeForce GTX 1080Ti 11 GB GPU (Lenovo, Bratislava, Slovakia). In addition, with the aid of Google Earth and Cesium, photogrammetry data can be easily imported into the UE interface, and the UAV dynamics and sensors are included using the AirSim plugin.

Once the set-up is finalized, a Python v3.6.0 file is used to establish a UDP connection between the Spirent GSS7000 hardware (Spirent PLC, Paignton, UK), and the hosting computer. Thus, a link between AirSim and the GSS7000 allows the recording of IMU data using the SimGEN v7.02 software. At the same time, RF signals are generated and sent further to the C009-F9P Ublox board (u-blox AG, Zürcherstrasse, Switzerland), which is responsible for processing the GNSS signals. By using OKTAL-SE Sim3D v4.7 in conjunction with Spirent’s GSS7000 hardware, the generated RF GNSS signals also include multipath effects. More details regarding the IMU and U-Blox F9P GNSS receiver can be viewed in Table 1.

## 5. Evaluation

### 5.1. Scenario Definition

**Figure 6:**
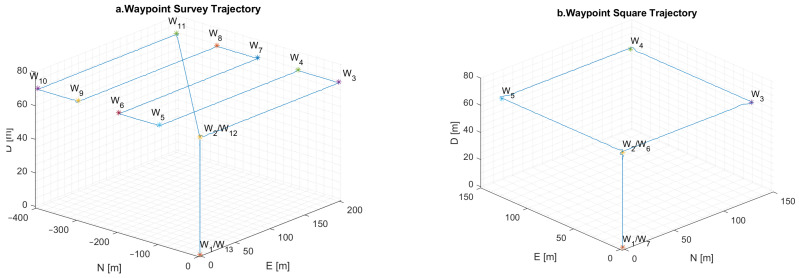
Waypoint survey trajectory—**left** (**a**); waypoint square trajectory—**right** (**b**).

To evaluate the hybrid FF architecture, Unreal Engine is implemented along with AirSim, as specified in Section 3. For this specific simulation, real photogrammetry data from the city center of Toulouse is integrated into Unreal Engine to replicate an urban environment. To further assess the fusion framework, two trajectories are used, as can be seen in Figure 6. The first trajectory is formed by different waypoints covering a larger area, while the second trajectory is limited to a 150-meter square area. Both trajectories are replicating a survey mission conducted in an urban environment. The simulation aims to test the VO algorithm while assessing the influence of multipath on the GNSS and the drift introduced by the MEMS IMU over time.

### 5.2. Light and Weather Evaluation

**Figure 7:**
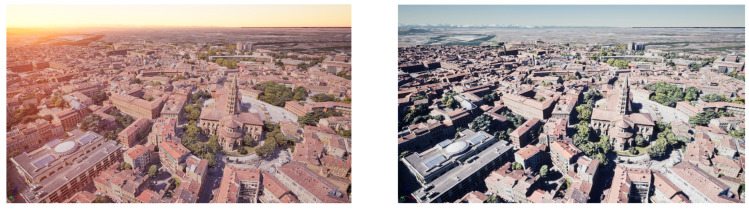
Toulouse in UE during the afternoon—**left**; Toulouse in UE during the evening—**right**.

**Figure 8:**
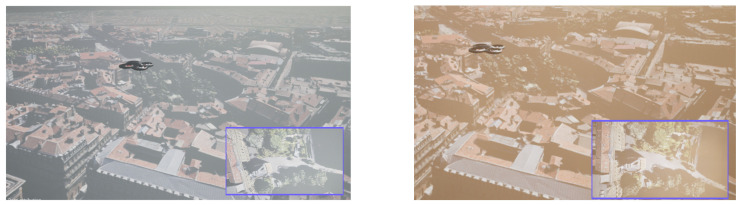
The UAV during fog operations—**left**; the UAV during dust operations—**right**.

In addition, both fusion architectures are tested under different light and weather conditions. The first scenario simulates the flight of the UAV during normal daylight conditions at 14:51 p.m. local time in Unreal Engine, while the second flight is at 18:00 p.m., as can be seen in Figure 7. Additionally, to further evaluate the accuracy of the VO algorithm, two more scenarios are considered, performing the two trajectories under fog and dust conditions, as can be seen in Figure 8.

### 5.3. Camera Calibration Set-Up

Considering the realism introduced into the simulation, before evaluating both fusion architectures, the monocular camera, rigidly fixed on the UAV and pointing downward, is configured in AirSim with an image width of 752 pixels and an image height of 480 pixels.

**Figure 9:**
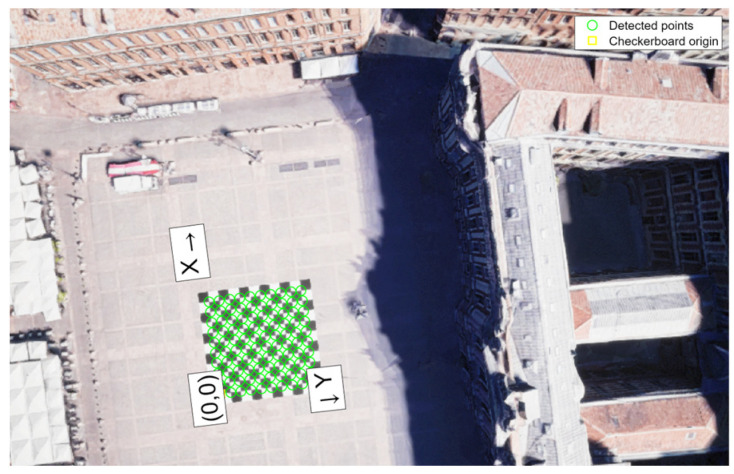
Calibration process for the monocular camera used in Unreal Engine.

Before using the monocular camera with the VO algorithm, a chessboard is introduced into Unreal Engine in order to calibrate the camera by finding the intrinsic matrix, defined as *K*, formed by the focal lengths $f_{x}$ and $f_{y}$ and by the optical centers $c_{x}$ and $c_{y}$. The chessboard is characterized by 10 rows and 10 columns, featuring alternating white and black squares, as can be seen in Figure 9. Each block has a dimension of 2 m in both length and width on the defined chessboard. Furthermore, in Unreal Engine, the UAV performed a small square trajectory over the chessboard at a cruise altitude of 70 m while recording all the camera frames. Then, the 220 frames extracted from Unreal Engine are processed using the MATLAB 2023b ‘Camera Calibrator’ toll, obtaining the intrinsic matrix, as defined in Equation (A3). Thus, the intrinsic matrix is used to enhance the realism of the VO algorithm.

(12) $$K=\left[\begin{array}{ccc} f_{x} & 0 & c_{x}\\ 0 & f_{y} & c_{y}\\ 0 & 0 & 1 \end{array}\right]=\left[\begin{array}{ccc} 378.6062 & 0 & 376.2584\\ 0 & 378.6560 & 240.4231\\ 0 & 0 & 1 \end{array}\right]$$

### 5.4. Conventional Federated Filter Architecture

**Figure 10:**
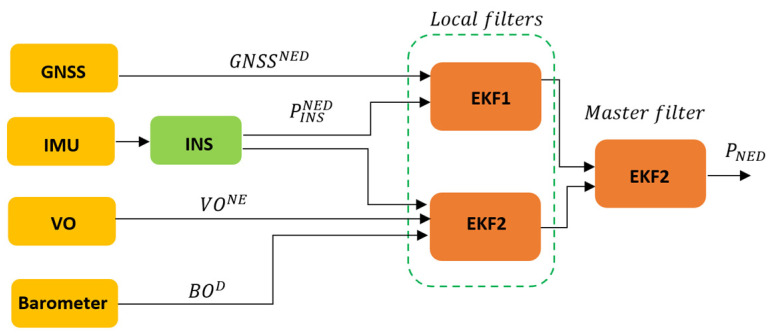
FF architecture with EKFs.

To compare the advantages of the proposed hybrid FF architecture, a conventional FF architecture formed only by EKFs is used, as can be seen in Figure 10, in a loosely coupled approach.

Thus, the INS block does not have any GRU model that predicts the IMU drift over time, and the master filter is only formed by a conventional EKF that fuses the positioning output from the two local filters. To further evaluate the FF, the same architecture is used, but while using the trained GRU model to enhance the INS position output.

### 5.5. Evaluation of the Square Trajectory

To evaluate the performance of both fusion architectures, various metrics are implemented, including 3D positioning, horizontal and vertical error, and RMSE (root mean square error) on each axis, as specified in Appendix B.

Although the pseudo-range accuracy of the GNSS receiver is 3 m as it can be seen in Table 1, the multipath introduced is consistently affecting the position accuracy of the first local filter when no GRU correction are used. As it can be seen in Table 2, for the square trajectory, significant positioning improvements can be observed in the first EKF by fusing the output from the GRU aid, which enhances the INS position output, with GNSS data, obtaining an equivalent horizontal error of 0.59 m (95th percentile). In contrast, the EKF without the GRU model shows a higher horizontal error of 9.76 m (95th percentile). Analysing the vertical error in both local filters, it can be observed that the filter without GRU corrections has the worst performance against the local filter with GRU corrections.

**Figure 11:**
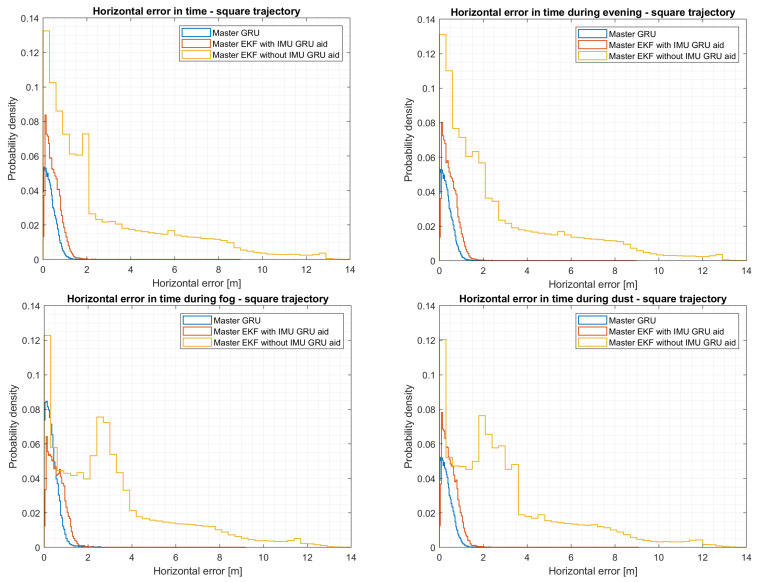
Horizontal error comparisons during different light conditions considering a square trajectory.

On the other hand, the second local filter, which fuses the output from the IMU with the VO and barometer data, shows slightly better performance with a horizontal error of 9.03 m (95th percentile) without relying on any corrections from the GRU model. Instead, when the IMU/GRU corrections are implemented, the horizontal error tends to achieve an equivalent positioning output of 4.57 m (95th percentile), with an improvement in the N, E, and D coordinates with an equivalent RMSE of 1.32 m, 1.45 m, and 0.76 m, respectively. The overall RMSE, considering all the NED coordinates, equals 2.11 m against the filter without GRU corrections, which equals 4.54 m. By analyzing the output of the master filters, it is possible to notice an enhancement in positioning, shifting from a horizontal error of 9.03 m (95th percentile) to 0.54 m (95th percentile) when employing the GRU model as the master filter instead of the master EKF. If a master EKF is considered with a GRU aid, slightly worse performances can be observed, maintaining a sub-meter horizontal error. By changing the light conditions from a daylight flight to an evening flight, in Table 2, it is possible to notice the influence of the VO over the output of the EKF. Despite the change in light conditions while executing the same trajectory, comparable performances are attained, as can be seen in Figure 11. Although comparable positioning performances are achieved during both afternoon and evening flights, a degradation in positioning is evident when weather conditions change from clear-sky conditions to the presence of fog and dust effects.

From Figure 11, it is possible to notice that the distribution of the horizontal error under fog and dust conditions is greater compared to the horizontal error observed in flights conducted during the afternoon and evening conditions. By analyzing the outputs of the second local EKF, which uses VO for positioning, from Table 3, it is possible to highlight that fog, in comparison to dust, degrades the EKF positioning output more, leading to a horizontal error of 14.25 m (95th percentile). In comparison, the dust effect leads to a horizontal error of 13.62 m (95th percentile). On the one hand, processing both fog and dust outputs with a master EKF leads to similar results due to the fusion with the output from the first local filter. On the other hand, the aid of the master GRU filter substantially improves the positioning output, decreasing the horizontal error to 0.57 m during fog conditions and 0.55 m during dust conditions. On the one hand, the master GRU model does cope with all the additional instabilities introduced by the VO during adverse and challenging weather conditions. In contrast, if a master EKF is used with a GRU aid, comparable results are obtained, as in the previous cases, during afternoon and evening flights. Thus, the VO algorithm can increase the overall position of an UAV in an urban environment during normal conditions, up to good light and weather conditions.

### 5.6. Evaluation of the Survey Trajectory

If a more complex trajectory is considered, it can be observed from Table 4 that the second local filter introduces more errors into the fusion system. This correlates to the drift introduced into the second EKF filter over time by the VO algorithm and INS. Considering that the survey trajectory covers a larger area, green areas such as parks decrease the efficiency of the VO algorithm due to the lack of features. Although slightly better performances are achieved when the VO output is fused with the output from the MEMS barometer and MEMS IMU with GRU corrections, the horizontal error is higher in comparison to the values presented in both Table 2 and Table 3. Although the perturbances of the VO algorithm are higher, it can be observed that both the master EKF and master GRU models substantially reduce the final positioning error.

**Figure 12:**
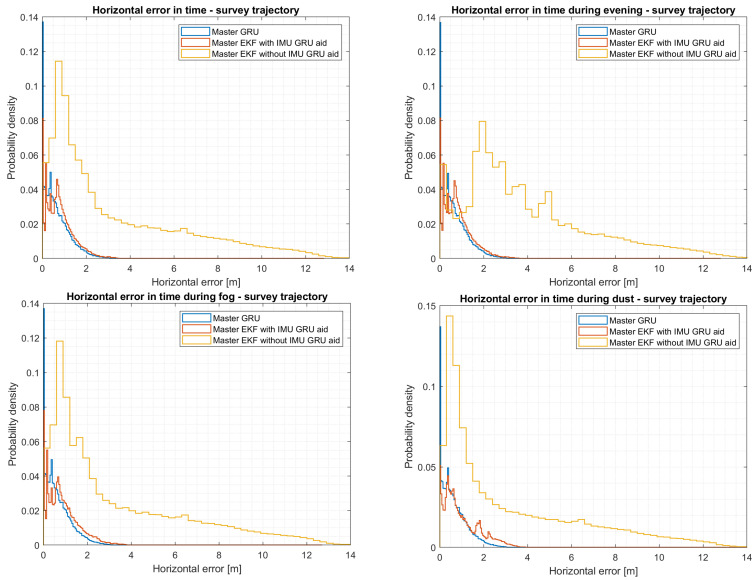
Horizontal error comparisons during different light and weather conditions considering a survey trajectory.

As can be seen in Figure 12, the master EKF has better performance during the afternoon flight, while the master EKF with data collected during evening conditions has more errors. In contrast, the master GRU model shows better performance, boasting a horizontal error of 1.72 m (95th percentile) and a vertical error of 0.28 m (95th percentile). Considering the effects of weather on the VO algorithm, as presented for the square trajectory, it can be seen from Figure 12 and Table 5 that more errors are introduced into the fusion framework during foggy conditions. However, in both situations, the trained GRU model, by filtering both fusion outputs and considering all the NED coordinates, achieves an RMSE of 0.83 m.

### 5.7. Evaluation of the Square and Survey Trajectories Considering GNSS Outages

**Figure 13:**
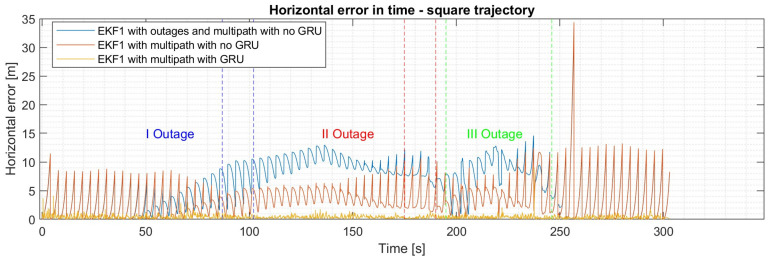
Horizontal error in time for the survey trajectory with multipath and outages over the EKF1.

To further investigate the performance of the proposed hybrid fusion architecture, GNSS outages are introduced into the simulation. For the square trajectory, two small outages lasting 15 seconds each are considered, along with the introduction of a more extended outage lasting 50 seconds. As can be seen from Table 6, the local EKF fusing data from the GNSS and IMU have an equivalent horizontal error of 12.16 m (95th percentile), which is higher in comparison to the previous scenario where outages were not considered, as can be seen in Figure 13. On the one hand, when the GNSS data are fused with the second local filter and the master EKF, an improvement in position can be seen, leading to a horizontal error of 9.44 m (95th percentile). Although the GNSS data is affected by multipaths and outages, it can be observed that the VO algorithm along the barometer from the second local EKF contains the errors introduced by GNSS outages. On the other hand, the GRU model used to predict INS errors substantially reduces the GNSS errors, leading to a horizontal error of 0.82 m (95th percentile), while the master GRU filter further reduces the horizontal error, improving it by 32%. Although similar performances are obtained during the evening flight scenario, it can be observed that due to the VO degradation in foggy and dusty conditions, the master EKF leads to a higher position error. In contrast, the trained master GRU filter achieves a sub-meter position error, despite the disturbances introduced during the simulation. Similar results are obtained using a master EKF with GRU corrections, leading to sub-meter accuracy with a horizontal error of 0.75 m (95th percentile) and a vertical error of 0.31 m (95th percentile).

**Figure 14:**
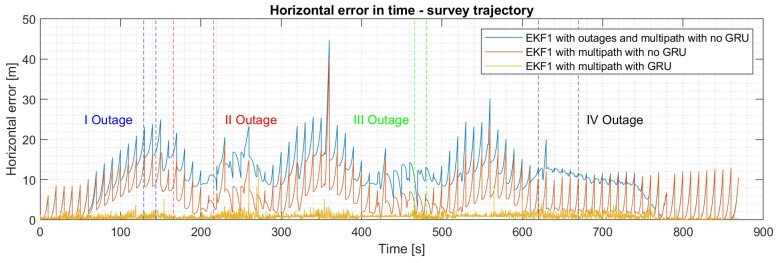
Horizontal error in time for the square trajectory with multipath and outages over the EKF1.

Instead, for the survey trajectory, two short GNSS outages lasting 15 seconds and two extended GNSS outages lasting 50 seconds are introduced into the simulation, as can be seen from Figure 14. The horizontal and vertical errors are degraded in comparison to the scenario where outages are not considered. At the same time, in the square scenario, the IMU, monocular camera, and MEMS barometer sensor reduce the errors introduced by the GNSS trying to cope with all the outages. It can be observed from Table 7 that even if a federated multi-sensor fusion framework is implemented, the master EKF cannot guarantee a reliable and stable flight in a multipath environment with outages. However, if a master GRU filter is used, the horizontal error tends to be within 2 m (95th percentile) despite all the external instabilities. Instead, if a master EKF filter is implemented with INS GRU corrections, the horizontal error tends to be within 4 m (95th percentile).

### 5.8. Performance Comparison

To evaluate the results obtained for the second local filter, which fuses the position output from the VO algorithm, the altitude from the MEMS barometer, and the position from the MEMS IMU, the following paper [48] is used as a benchmark. The paper implements a VINS-mono [49] algorithm that gathers real data from a monocular camera mounted on a UAV pointing downward. It can be observed that the output from the second local filter shows significant improvements at 60 m while considering both the square and survey trajectories, with an equivalent improvement of 61.78% for the square scenario and 17.89% for the survey trajectory. It can be observed that with longer trajectories, the errors introduced by the VO accumulate over time, decreasing the position accuracy.

Instead, to compare the overall performances obtained by the proposed hybrid federated fusion architecture, the solution presented in [50] is used as a comparison. The paper presents a robust adaptive Kalman filter for gathering data from a GNSS sensor, an IMU MEMS sensor, and an optical camera. As observed in Table 8, the proposed solution with an ML aid shows better performances considering both scenarios during normal VO operations and with multipaths.

## 6. Conclusions

In this study, the authors presented and demonstrated the following:

- A novel hybrid sensor fusion framework based on a federated approach was developed and tested in a loosely coupled set-up, integrating data from diverse sources, including a GNSS receiver, a MEMS IMU sensor, a monocular camera, and a MEMS barometer.
- A virtual environment was developed in UE, along with AirSim, Cesium, and photogrammetry data imported from Google Earth, allowing the authors to test and validate the effects of the VO algorithm over the hybrid fusion framework under different light and weather conditions. To further validate the framework, the hybrid FF architecture was compared to a classic FF framework. GNSS data were enhanced using the Spirent GSS7000 simulator with the OKTAL-SE Sim 3D software stack, introducing multipath during the data collection phase, and collected using a C009-F9P Ublox board. At the same time, IMU data were gathered using the Spirent GSS7000. In addition, GNSS outages were considered for both scenarios.
- Based on the performance metrics presented in Table 2 and Table 3 for a square trajectory and Table 4 and Table 5 for a survey trajectory, it is evident that the corrections offered by the master GRU model surpass those of the master EKF filter. The master GRU model demonstrates the capability to achieve a sub-meter positioning error in terms of horizontal and vertical error for the square trajectory and below 2 m for the survey trajectory, under different weather and light conditions.
- The presented feature-based VO algorithm does improve the position accuracy of the UAV, as can be seen in Table 2 and Table 3 under good weather and light conditions. If more complex and longer missions are considered, the VO algorithm does not provide major position correction.

## Figures and Tables

**Table 1:** INS and U-Blox specifications.

Sensor Specifications					
Accelerometer		Gyroscope		U-Blox F9P GNSS Receiver Specification	
Scaling factor (ppm)	500	Scaling factor (ppm)	500	Pseudo-range accuracy (m)	3
Bias (mg)	0.1	Bias (deg/h)	0.001	Pseudo-range rate accuracy (m/s)	0.5
ARW (m/s/sqrt(h))	0.003	GRW (deg/sqrt(h))	0.003	Update rate (Hz)	1
Update rate (Hz)	100			-	

**Table 2:** Positioning performance for Toulouse under different light conditions—square trajectory.

Toulouse—Afternoon							
Position Source	3D Position Error (95th Percentile) [m]	Horizontal Error (95th Percentile) [m]	Vertical Error (95th Percentile) [m]	RMSE N [m]	RMSE E [m]	RMSE D [m]	RMSE NED [m]
EKF1 IMU/GNSS (no GRU aid)	10.40	9.76	3.5	4.49	1.35	3.36	5.77
EKF2 IMU/VO/BO (no GRU aid)	9.09	9.03	1.58	4.17	1.60	0.78	4.54
Master EKF filter (no GRU aid)	9.09	9.03	1.50	4.18	1.35	0.77	4.46
EKF1 IMU/GNSS (with GRU aid)	0.64	0.59	0.29	0.20	0.17	0.13	0.30
EKF2 IMU/VO/BO (with GRU aid)	4.57	4.57	1.48	1.32	1.45	0.76	2.11
Master GRU filter (with GRU aid)	0.58	0.54	0.27	0.16	0.14	0.12	0.25
Master EKF filter (with GRU aid)	0.76	0.73	0.29	0.21	0.21	0.13	0.33
Toulouse—Evening							
EKF2 IMU/VO/BO (no GRU aid)	9.16	9.00	2.54	4.17	1.64	1.40	4.68
Master EKF filter (no GRU aid)	9.13	8.95	2.44	4.15	1.63	1.37	4.58
EKF2 IMU/VO/BO (with GRU aid)	5.07	4.92	1.63	1.52	1.58	1.00	2.35
Master GRU filter (with GRU aid)	0.59	0.54	0.27	0.16	0.14	0.12	0.25
Master EKF filter (with GRU aid)	0.77	0.75	0.29	0.22	0.21	0.13	0.34

**Table 3:** Positioning performance for Toulouse under different weather conditions—square trajectory.

Toulouse—Fog							
Position Source	3D Positioning Error (95th Percentile) [m]	Horizontal Error (95th Percentile) [m]	Vertical Error (95th Percentile) [m]	RMSE N [m]	RMSE E [m]	RMSE D [m]	RMSE NED [m]
EKF2 IMU/VO/BO (no GRU aid)	14.63	14.25	3.00	6.34	5.07	1.60	8.28
Master EKF filter (no GRU aid)	9.44	8.96	2.98	4.12	1.71	1.60	4.74
EKF2 IMU/VO/BO (with GRU aid)	8.63	8.63	3.25	2.80	2.53	1.48	4.07
Master GRU filter (with GRU aid)	0.61	0.57	0.28	0.17	0.17	2.30	0.27
Master EKF filter (with GRU aid)	0.89	0.87	0.29	0.25	0.24	0.13	0.37
Toulouse—Dust							
EKF2 IMU/VO/BO (no GRU aid)	13.74	13.62	4.90	6.54	3.90	2.54	8.03
Master EKF filter (no GRU aid)	9.65	8.82	4.85	4.09	1.57	2.50	5.06
EKF2 IMU/VO/BO (with GRU aid)	6.00	5.51	2.50	1.77	1.61	1.45	2.80
Master GRU filter (with GRU aid)	0.60	0.55	0.27	0.16	3.07	0.12	0.25
Master EKF filter (with GRU aid)	0.79	0.77	0.29	0.22	0.22	0.13	0.34

**Table 4:** Positioning performance for Toulouse under different light conditions—survey trajectory.

Toulouse—Afternoon							
Position Source	3D Positioning Error (95th Percentile) [m]	Horizontal Error (95th Percentile) [m]	Vertical Error (95th Percentile) [m]	RMSE N [m]	RMSE E [m]	RMSE D [m]	RMSE NED [m]
EKF1 IMU/GNSS (no GRU aid)	11.37	10.19	4.05	1.77	4.52	3.77	6.15
EKF2 IMU/VO (no GRU aid)	22.92	22.90	5.00	9.38	7.36	1.60	12.03
Master EKF filter (no GRU aid)	10.44	10.19	2.11	1.94	4.48	1.80	5.21
EKF1 IMU/GNSS (with GRU aid)	1.86	1.85	0.26	0.76	0.49	0.16	0.92
EKF2 IMU/VO (with GRU aid)	11.74	11.74	4.10	4.79	4.63	1.27	6.79
Master GRU filter (with GRU aid)	1.72	1.72	0.28	0.67	0.44	0.12	0.81
Master EKF filter (with GRU aid)	1.93	1.93	0.26	0.77	0.53	0.16	0.95
Toulouse—Evening							
EKF2 IMU/VO (no GRU aid)	23.43	23.43	5.53	10.00	7.55	1.64	12.64
Master EKF filter (no GRU aid)	10.57	10.40	5.31	2.71	4.48	1.57	5.47
EKF2 IMU/VO (with GRU aid)	13.38	13.32	3.99	5.27	5.46	1.73	7.79
Master GRU filter (with GRU aid)	1.73	1.73	0.28	0.67	0.16	0.12	0.82
Master EKF filter (with GRU aid)	1.97	1.96	0.26	0.78	0.54	0.16	0.97

**Table 5:** Positioning performance for Toulouse under different weather conditions—survey trajectory.

Toulouse—Fog							
Position Source	3D Positioning Error (95th Percentile) [m]	Horizontal Error (95th Percentile) [m]	Vertical Error (95th Percentile) [m]	RMSE N [m]	RMSE E [m]	RMSE D [m]	RMSE NED [m]
EKF2 IMU/VO (no GRU aid)	23.76	23.76	10.44	9.10	7.95	3.01	12.50
Master EKF filter (no GRU aid)	11.36	10.21	10.31	1.80	4.52	3.10	5.77
EKF2 IMU/VO (with GRU aid)	25.22	25.19	8.34	10.39	9.92	2.80	14.64
Master GRU filter (with GRU aid)	1.74	1.73	0.29	0.67	0.46	0.13	0.83
Master EKF filter (with GRU aid)	2.22	2.21	0.26	1.07	0.87	0.60	1.07
Toulouse—Dust							
EKF2 IMU/VO (no GRU aid)	36.78	36.75	8.04	14.83	10.24	2.77	18.24
Master EKF filter (no GRU aid)	11.32	10.18	3.97	1.84	4.5	3.71	6.12
EKF2 IMU/VO (with GRU aid)	22.51	22.12	5.95	8.73	9.12	4.08	13.27
Master GRU filter (with GRU aid)	1.74	1.73	0.29	0.67	0.46	0.13	0.83
Master EKF filter (with GRU aid)	2.53	2.53	0.26	10.03	0.56	0.16	1.18

**Table 6:** Positioning performance for Toulouse under different light conditions with GNSS outages—square trajectory.

Toulouse—Afternoon							
Position Source	3D Position Error (95th Percentile) [m]	Horizontal Error (95th Percentile) [m]	Vertical Error (95th Percentile) [m]	RMSE N [m]	RMSE E [m]	RMSE D [m]	RMSE NED [m]
EKF1 IMU/GNSS (no GRU aid)	15.81	12.16	11.73	5.19	5.51	7.22	13.92
Master EKF filter (no GRU aid)	9.48	9.44	1.52	4.03	2.23	0.82	6.01
EKF1 IMU/GNSS (with GRU aid)	0.98	0.82	0.59	0.34	0.33	0.34	1.01
Master GRU filter (with GRU aid)	0.60	0.55	0.27	0.17	0.18	0.12	0.28
Master EKF filter (with GRU aid)	0.80	0.75	0.31	0.23	0.23	0.17	0.37
Toulouse—Evening							
Master EKF filter (no GRU aid)	9.81	9.59	2.74	4.43	4.04	1.49	6.18
Master GRU filter (with GRU aid)	0.60	0.56	0.28	0.18	0.17	0.15	0.29
Master EKF filter (with GRU aid)	0.81	0.76	0.31	0.23	0.23	0.17	0.37
Toulouse—Fog							
Master EKF filter (no GRU aid)	19.66	19.44	2.94	8.29	7.59	1.65	11.36
Master GRU filter (with GRU aid)	0.86	0.81	0.43	0.28	0.17	0.16	0.37
Master EKF filter (with GRU aid)	0.92	0.88	0.31	0.25	0.26	0.17	0.40
Toulouse—Dust							
Master EKF filter (no GRU aid)	19.29	19.07	2.94	8.14	7.40	1.65	11.12
Master GRU filter (with GRU aid)	0.62	0.58	0.28	0.19	0.17	0.15	0.30
Master EKF filter (with GRU aid)	0.73	0.83	0.31	0.23	0.23	0.17	0.37

**Table 7:** Positioning performance for Toulouse under different light conditions with GNSS outages—survey trajectory.

Toulouse—Afternoon							
Position Source	3D Position Error (95th Percentile) [m]	Horizontal Error (95th Percentile) [m]	Vertical Error (95th Percentile) [m]	RMSE N [m]	RMSE E [m]	RMSE D [m]	RMSE NED [m]
EKF1 IMU/GNSS (no GRU aid)	17.83	17.83	13.16	6.34	8.91	10.85	15.41
Master EKF filter (no GRU aid)	17.82	17.78	4.99	6.35	8.88	1.59	11.03
EKF1 IMU/GNSS with GRU aid	3.39	3.35	0.60	1.71	0.83	0.49	1.98
Master GRU filter (with GRU aid)	1.77	1.77	0.27	0.49	0.69	0.13	0.86
Master EKF filter (with GRU aid)	3.42	3.37	0.60	1.68	0.87	0.49	1.96
Toulouse—Evening							
Master EKF filter (no GRU aid)	17.79	17.75	5.51	6.38	8.85	1.63	11.03
Master GRU filter (with GRU aid)	1.77	1.76	0.27	0.49	0.69	0.13	0.86
Master EKF filter (with GRU aid)	3.43	3.39	0.60	1.69	0.87	0.49	1.96
Toulouse—Fog							
Master EKF filter (no GRU aid)	17.87	17.74	10.64	6.35	8.85	3.20	11.36
Master GRU filter (with GRU aid)	1.76	1.76	0.27	0.49	0.69	0.13	0.86
Master EKF filter (with GRU aid)	3.50	3.46	0.60	1.69	0.89	0.49	1.98
Toulouse—Dust							
Master EKF filter (no GRU aid)	18.01	17.81	8.02	6.50	8.89	2.77	11.36
Master GRU filter (with GRU aid)	1.76	1.76	0.27	0.49	0.69	0.13	0.86
Master EKF filter (with GRU aid)	3.48	3.44	0.60	1.69	0.89	0.49	1.97

**Table 8:** Benchmark analysis.

Algorithm	MAE N [m]	MAE E [m]	MAE D [m]	RMSE NED [m]	Square Scenario		Survey Scenario	
Mono-VIO [48]	-	-	-	16.54	61.78%		17.89%	
Robust Adaptive Kalman filter [50]	0.06	0.07	0.06	-	N	193%	N	188%
					E	180%	E	192%
					D	187%	D	199%

**Figure A1:**
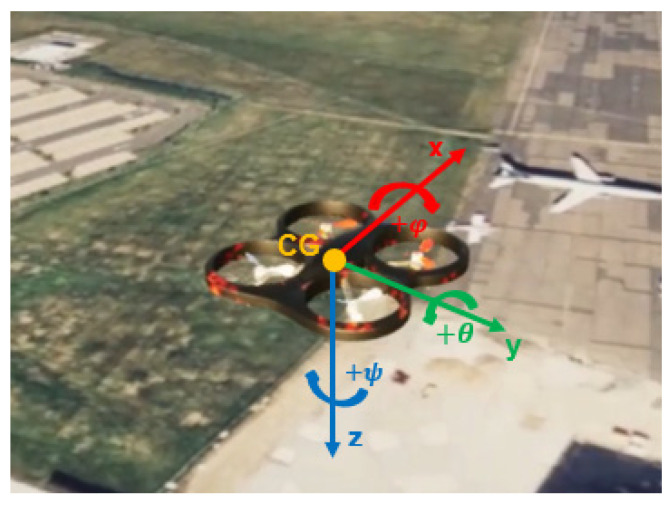
UAV’s body frame definition in AirSim.

## Data Availability

Data are contained within the article.

## Appendix A. Coordinate Systems Definition and Transformations

### Appendix A.1. Body Frame

The first coordinate system is defined by the body frame having its origin in the UAV’s center of gravity, where the x axis points ahead, the z axis downwards, and the y axis on the right side of the flying vehicle, while rotation on the x axis is defined as φ (roll), rotation on the y axis as θ (pitch), and rotation on the z axis as ψ (yaw), as can be seen in the figure below.

### Appendix A.2. ECEF (Earth-Centered Earth-Fixed Frame)

The Earth-Centered Earth-Fixed Frame has its origin at the Earth’s center, and it is fixed and rotates with the Earth. The x axis points to the prime meridian, the z axis points towards the North Pole, and the y axis completes the right-hand frame.

### Appendix A.3. Inertial Frame

The inertial frame aligns with the Earth’s center, similar to the ECEF frame, except its rotation is independent. The x axis points towards the mean vertical equinox, the z axis is parallel with the Earth’s rotation, and the y axis completes the right-handed frame, similar to the ECEF frame.

### Appendix A.4. Navigation Frame

A NED (North, East, and Down) [51] navigation coordinate frame attached to the vehicle is adopted by the author. Thus, the x axis points towards north (N), the z axis points downwards (D), and the y axis points east (E), considering a WGS84 ellipsoid Earth model.

### Appendix A.5. Conversion from WGS84 to ECEF

(A1) $$x_{ECEF}=\left(N+h\right)\cos\varphi \cos\lambda \;y_{ECEF}=\left(N+h\right)\cos\varphi \sin\lambda \;z_{ECEF}=\left[N\left(1-e^{2}\right)+h\right]\sin\varphi$$

$$N=\frac{a}{\sqrt{1-e^{2}{\sin\varphi }^{2}}}\;e^{2}=2f-f^{2}\;f=\frac{a-b}{a}\;P_{ECEF}=\left[\begin{array}{c} x_{ECEF}\\ y_{ECEF}\\ z_{ECEF} \end{array}\right]$$

where $x_{ECEF}$, $y_{ECEF}$, and $z_{ECEF}$ are the positions in the ECEF frame; φ, λ, and h are the initial altitude, longitude, and altitude, respectively; *N* is the radius curvature in prime vertical; *a* is the semi-major Earth axis; *b* is the semi-minor Earth axis; $e^{2}$ is the eccentricity of Earth; and *f* is the flattening.

### Appendix A.6. Conversion from ECEF to NED

(A2) $$P_{NED}=R\left(P_{ECEF}-P_{REF}\right)$$

$$R=\left[\begin{array}{ccc} -sin\varphi_{REF}cos\lambda_{REF} & -\;sin\varphi_{REF}sin\lambda_{REF} & cos\varphi_{REF}\\ -sin\lambda_{REF} & cos\lambda_{REF} & 0\\ -cos\varphi_{REF}cos\lambda_{REF} & -cos\varphi_{REF}sin\lambda_{REF} & -sin\varphi_{REF} \end{array}\right]\;x_{REF}=\left(N+h_{REF}\right)\cos\varphi_{REF}\cos\lambda_{REF}\;y_{REF}=\left(N+h_{REF}\right)\cos\varphi_{REF}\sin\lambda_{REF}\;z_{REF}=\left[N\left(1-e^{2}\right)+h\right]\sin\varphi_{REF}$$

where $P_{REF}$ represents the vector with the initial LLA coordinates in an ECEF coordinate system.

### Appendix A.7. Coordinate Frame Transformations

To convert the body frame defined previously to the NED frame, a DCM (direction cosine matrix) is used, as follows:

(A3) $$C_{b}^{n}=\left[\begin{array}{ccc} cos\theta \;cos\psi & -\;cos\varphi \;sin\psi +sin\varphi \;sin\theta \;cos\psi & sin\varphi \;sin\psi +cos\varphi \;sin\theta \;cos\psi \\ cos\theta \;sin\psi & cos\varphi \;cos\psi +sin\varphi \;sin\theta \;sin\psi & -sin\varphi \;cos\psi +cos\varphi \;sin\theta \;sin\psi \\ -sin\theta & sin\varphi \;cos\theta & cos\varphi \;cos\theta \end{array}\right]$$

where $C_{b}^{n}$ is the DCM matrix and θ ,ψ, and φ are the pitch, yaw, and roll angle in radians, respectively.

## Appendix B. Metrics

To assess the two fusion architectures, the following metrics were used:

(A4) $$\mathrm{Vertical}\;\mathrm{error}=\sqrt{{\left(D_{i}-{\hat{D}}_{i}\right)}^{2}}$$

(A5) $$\mathrm{Horizontal}\;\mathrm{error}=\sqrt{{\left(N_{i}-{\hat{N}}_{i}\right)}^{2}+{\left(E_{i}-{\hat{E}}_{i}\right)}^{2}}$$

(A6) $$3D\;\mathrm{error}=\sqrt{{\left(N_{i}-{\hat{N}}_{i}\right)}^{2}+{\left(E_{i}-{\hat{E}}_{i}\right)}^{2}+{\left(D_{i}-{\hat{D}}_{i}\right)}^{2}}$$

(A7) $$R{MSE}_{N}=\sqrt{\frac{1}{n}\sum_{i}^{n}{\left(N_{i}-{\hat{N}}_{i}\right)}^{2}}=\sqrt{\frac{1}{n}\sum_{i}^{n}{∆N_{i}}^{2}}\;{RMSE}_{E}=\sqrt{\frac{1}{n}\sum_{i}^{n}{\left(E_{i}-{\hat{E}}_{i}\right)}^{2}}=\sqrt{\frac{1}{n}\sum_{i}^{n}{∆E_{i}}^{2}}\;{RMSE}_{D}=\sqrt{\frac{1}{n}\sum_{i}^{n}{\left(D_{i}-{\hat{D}}_{i}\right)}^{2}}=\sqrt{\frac{1}{n}\sum_{i}^{n}{∆D_{i}}^{2}}$$

(A8) $${RMSE}_{NED}=\sqrt{{{RMSE}_{N}}^{2}+{{RMSE}_{E}}^{2}+{{RMSE}_{D}}^{2}}=\sqrt{\frac{1}{n}\sum_{i}^{n}\left({∆N_{i}}^{2}+{∆E_{i}}^{2}+{∆D_{i}}^{2}\right)}$$

where $N_{i}$, $E_{i}$, and $D_{i}$ are the ground truth positioning output from Unreal Engine in NED coordinates, while ${\hat{N}}_{i}$, ${\hat{E}}_{i}$, and ${\hat{D}}_{i}$ are the estimated NED position.
